# Morbidity Pattern in Young Unmarried Women Attending Gynecology Clinics at King Abdulaziz Medical City in Riyadh, Saudi Arabia

**DOI:** 10.7759/cureus.86088

**Published:** 2025-06-15

**Authors:** Mohammed A AlAteeq, Razan Al Kahtani, Shumukh AlDawsari, Sana Balghonaim, Zina AlMasri, Haneen Altuwaijri

**Affiliations:** 1 Family Medicine, Ministry of National Guard - Health Affairs, Riyadh, SAU; 2 Family Medicine, King Abdullah International Medical Research Center, King Saud bin Abdulaziz University for Health Sciences, Riyadh, SAU; 3 College of Medicine, King Saud bin Abdulaziz University for Health Sciences, Riyadh, SAU

**Keywords:** adolescents, dysmenorrhea, menstrual disorders, reproductive health, women health

## Abstract

Background: Gynecological morbidities, particularly menstrual disorders, are a leading cause of healthcare consultations among young, unmarried women globally. However, comprehensive data on their prevalence and treatment-seeking behaviors in this demographic, especially in Saudi Arabia, are limited. This study aimed to characterize the pattern of gynecological morbidities among young unmarried women attending clinics at King Abdulaziz Medical City (KAMC), Riyadh, Saudi Arabia.

Methods: A cross-sectional study analyzed medical records of 195 unmarried women aged 14-25 years presenting with gynecological issues at KAMC between January 1, 2018, and December 31, 2022. Data collected included demographics, BMI, comorbidities, gynecological and surgical history, chronic medications, presenting complaints, complaint duration, diagnoses, and management plans.

Results: Of the 195, 110 participants (56.41%) had at least one medical condition. The most frequent complaint was of irregular menstrual cycle, reported by 51 patients (26.2%). Polycystic ovary syndrome (PCOS) was diagnosed in 76 patients (39%), followed by ovarian masses. The most frequent management plan involved further investigations in 68 cases (34.9%), followed by oral contraceptive pill prescriptions. A significant association was found between the BMI category and PCOS diagnosis.

Conclusion: This study provides insight into gynecological health disparities among young unmarried women in Saudi Arabia. The findings highlight the high prevalence of comorbidities and PCOS within this cohort. Understanding these patterns can inform policy changes and resource allocation, ultimately improving gynecological healthcare delivery and outcomes for young unmarried women in Saudi Arabia.

## Introduction

Gynecological morbidities encompass a range of conditions affecting the woman's reproductive tract that are unrelated to pregnancy, abortion, or childbirth. These can include but are not limited to, reproductive tract infections, cervical cellular changes, pelvic organ prolapse, and urinary tract infections (UTIs) [1]. Menstrual disorders are also a primary reason for women seeking medical consultation globally, posing particular challenges for young, unmarried women [2,3]. Common menstrual abnormalities include dysmenorrhea [4,5], excessive bleeding [6], and abdominal pain [2]. Unfortunately, a significant stigma often discourages women from seeking gynecological treatment, as their complaints may be erroneously linked to sexually transmitted diseases, leading to social ostracization [7].

Well-woman visits are crucial for preventing gynecological and menstrual abnormalities [8]. These visits aim to identify modifiable risk factors and address concerns that could negatively impact a patient's overall health and well-being. Gynecological symptoms manifest differently across age groups. Studies from various regions have reported a high prevalence of dysmenorrhea among young, unmarried women [9,10]. Furthermore, research indicates that young women who seek timely treatment for gynecological issues experience fewer long-term abnormalities compared to those who delay or avoid care. Factors influencing a young woman's decision to seek treatment can include tribal status, educational attainment, and proximity to medical facilities [11].

Previous studies highlight the challenges surrounding gynecological health and treatment-seeking behavior in young women. A cross-sectional survey of female medical students at King Abdulaziz University in Jeddah, Saudi Arabia, revealed that 48.2% experienced irregular menstruation during exam periods. Dysmenorrhea was the most prevalent irregularity (70.9%), followed by prolonged cycles (45.6%) and excessive bleeding (41.9%). While 93% reported premenstrual symptoms, and 60.4% used various remedies for menstrual irregularities, 12.1% reported missing classes due to these issues. However, this study's sample was limited to university students, and diagnoses were not physician-confirmed, with a majority opting for self-management rather than professional medical intervention [6]. Similarly, a study in Mansoura, Egypt, involving 664 female secondary school students, found that approximately 75% reported dysmenorrhea (55.3% mild, 30.0% moderate, 14.8% severe) [5]. Despite this, most students did not seek medical help, with 34.7% self-treating [5]. Another study on treatment-seeking behavior in Jeddah, Saudi Arabia, among 1931 high school students, reported that 83% had dysmenorrhea, 34% irregular menstruation, and 27% heavy menstrual bleeding; yet, only 23% sought medical help for their menstrual problems [12]. Notably, reports of heavy menstrual bleeding in this study were subjective and lacked physician diagnosis [12]. A 2018 study at King Khalid University in Saudi Arabia indicated that two-thirds of participants (70.6% of young women) complained of dysmenorrhea, but only 23% consulted a doctor [13]. Like other regional studies, the sample consisted of university students, limiting generalizability to all young Saudi women [13]. Many adolescents with menstrual problems avoid seeking treatment due to embarrassment, fear of illness, and lack of awareness regarding available treatments, with irregular menstruation and dysmenorrhea being the most common issues [14]. A 2016 study in Bihar and Uttar Pradesh, India, on adolescent girls aged 10-19, found that while roughly a quarter (23.6%) suffered from gynecological morbidities, only a third of these girls sought medical treatment [11]. Factors increasing treatment-seeking likelihood included higher education (10+ years), non-caste/scheduled tribe status, and urban residency [11]. Furthermore, a survey of non-pregnant women in Odisha, India, concluded that while married women sought treatment for reproductive tract infection symptoms, unmarried younger women were less likely to do so due to insufficient awareness [15].

To our knowledge, there is a significant dearth of data concerning the patterns of gynecological morbidities and treatment-seeking behaviors among young, unmarried women both locally and regionally. Given that young, unmarried women represent an underserved demographic in Saudi Arabia concerning gynecological care, this study aims to describe the morbidity patterns of this population attending a gynecology clinic at King Abdulaziz Medical City in Riyadh, Saudi Arabia.

## Materials and methods

This cross-sectional study was conducted at the Outpatient Department (OPD) of the Gynecology Department at King Abdulaziz Medical City-Riyadh, National Guard, Riyadh, Saudi Arabia. This department is characterized by a high patient volume, with an average of 11,000 new patients annually. It comprises four divisions, offering 149 beds for admission, 127 beds for ward admissions and induction units, and seven beds for assessment rooms. The study population included 195 unmarried Saudi women aged 14-25 years. Exclusion criteria encompassed non-Saudi individuals, married women, and those presenting with non-gynecological complaints.

The Institutional Review Board (IRB), King Abdullah International Medical Research Center (KAIMRC), Riyadh issued approval (IRB/21209/23) for the study.

Data collection process

Data were collected by reviewing the electronic medical records (BestCare) system of eligible participants for gynecology-related visits between January 1, 2018, and December 31, 2022. Data extraction was performed by trained research assistants using a pre-designed, standardized data collection sheet. This sheet was structured into four sections, including the following variables: age, medical background (chronic medical conditions, chronic medications, past gynecological, and surgical history), and gynecology-related visit details (complaint, duration, diagnosis, and management plan).

To ensure data accuracy and consistency, several measures were implemented. Before data extraction began, research assistants underwent standardized training on the precise definition and coding of each variable as outlined in the data collection sheet.

Data validation checks were performed during and after the extraction process. Any inconsistencies or apparent errors (e.g., illogical values, conflicting entries) were identified and resolved by revisiting the original electronic medical record in BestCare (ezCaretech Co., Ltd, Seoul, South Korea).

Data points that were genuinely absent from the electronic medical record, including "complaint duration," were meticulously recorded as "not available" (N/A). The frequency of these missing entries was considered a significant observation, highlighting potential deficiencies in the comprehensiveness of the electronic health record system's documentation practices.

Chronic medical conditions and chronic medications were primarily categorized based on systems or major conditions. Conditions and medications that did not fit into these predefined primary categories, or those with very low frequencies that precluded meaningful individual analysis, were grouped into an "Other" category. This aggregation ensured that all recorded data were captured while maintaining a manageable number of variables for statistical analysis. For "Other Health Conditions," this category included diagnoses such as alopecia and acne. Similarly, "Other Medications" encompassed substances like salbutamol, Plaquenil, and cyclosporine. While not exhaustive, these examples illustrate the diverse nature of items within these aggregated categories.

Data analysis

Collected data were initially entered into Microsoft Excel 2019 (Microsoft Corp., Redmond, WA, USA) and subsequently transferred to IBM SPSS for Windows, Version 27 (IBM Corp., Armonk, NY, USA) for statistical analysis. Continuous variables (age and BMI) were presented as mean ± standard deviation (SD). Mutually exclusive categorical data (e.g., duration of complaint) were encoded using a one-step process, assigning a unique numerical value to each variable, and then presented as frequency tables and percentages. Mutually inclusive categorical data (e.g., comorbidities, surgical history, gynecological history, chronic medication, complaint, diagnosis, and management) underwent a two-step encoding process. First, each unique variable received a numerical assignment, followed by a second encoding to reflect determined categories. These data were also presented as frequency tables and percentages. The prevalence of abnormalities was determined with a 95% confidence interval (CI). For inferential statistics, BMI was converted from a continuous to a categorical variable (underweight, normal, overweight, obese) and compared to diagnosis categories using a Chi-square test.

## Results

A total of 195 patients were included in the study analysis. The participants had a mean age of 19.59 ± 3.72 years and a mean BMI of 25.63 ± 6.74 kg/m. Distribution by BMI category revealed that 76 (38.97%) were of normal weight, 54 (27.69%) were overweight, 41 (21.03%) were obese, and 24 (12.31%) were underweight.

Table 1 summarizes patient medical conditions, chronic medication use, and gynecological and surgical histories. Of the 195 patients, 110 (56.41%) presented with at least one condition. Furthermore, 68 (34.9%) reported concurrent use of at least one chronic medication. A significant majority, 146 (74.9%), had no history of gynecological illness. Among patients with a positive gynecological history (n=49), polycystic ovary syndrome (PCOS) was the most prevalent diagnosis found in 31 patients (63.27%), followed by ovarian masses/cysts in six patients (12.24%), primary dysmenorrhea in five patients (10.2%), and abnormal uterine morphology (bicornuate, absent uterus) in four patients (8.16%). Forty-one patients (21%) had a history of previous surgical intervention. Of these, 32 patients (78.05%) had undergone at least one non-gynecological procedure, with the remainder having a history of at least one gynecological procedure.

**Table 1:** Risk factor profile of comorbidities, chronic medications, gynecological and surgical histories among patients * Patients may possess multiple co-morbidities, gynecological history types, surgical history types, and take several chronic medications

Variables*	n (%)
Presence of medical conditions	110 (56.41%)
Cardiovascular	5 (2.6%)
Hematological	35 (17.9%)
Endocrine	27 (13.8%)
Psychological	10 (5.1%)
Neurological	7 (3.6%)
Musculoskeletal	4 (2.1%)
Respiratory	7 (3.6%)
Nephrological	6 (3.1%)
Developmental	7 (3.6%)
Other	40 (20.5%)
N/A	85 (43.6%)
Chronic medication usage	68 (34.9%)
Oral contraceptive pill (OCP)	14 (7.18%)
Anti-diabetic	6 (3.1%)
Anti-hypertensive	4 (2.1%)
Tranexamic acid	3 (1.5%)
Iron supplement	4 (2.1%)
Psychiatric medications	7 (3.6%)
Other	42 (21.54%)
N/A	127 (65.1%)
Presence of gynecological disease	49 (25.1%)
Primary dysmenorrhea	5 (2.6%)
Polycystic ovary syndrome (PCOS)	31 (15.9%)
Ovarian mass/cyst	6 (3.1%)
Abnormal uterus (bicornuate, absent)	4 (2.1%)
Breast disease/lump	2 (1.0%)
Pelvic inflammatory disease (PID)	2 (1.0%)
Ovarian failure	1 (0.5%)
Other	3 (1.5%)
N/A	146 (74.9%)
Presence of surgical history	41 (21%)
Gynecological surgeries	14 (7.18%)
Other	32 (16.41%)
N/A	127 (65.1%)

Table 2 provides a comprehensive overview of patient complaints, complaint duration, diagnoses, and management strategies. The most frequently reported complaint was irregular menstrual cycles reported by 51 patients, accounting for 26.2% of presentations, followed by amenorrhea in 30 patients (15.4%) and menorrhagia in 27 patients (13.8%). Regarding symptom duration, 12 patients (6.2%) reported onset since menarche, 32 patients (16.4%) indicated a duration of less than six months, 26 patients (13.3%) between six months and one year, and 14 patients (7.2%) greater than one year. Notably, the duration of the complaint was undocumented for the largest subgroup, comprising 111 patients (56.9%). Polycystic ovary syndrome (PCOS) was the predominant diagnosis within the sample population, found in 76 patients and accounting for 39% of cases. Ovarian cysts were diagnosed in 22 patients (11.3%), while 29 cases (14.9%) remained undiagnosed at the time of data collection. Management plans for the 195 patients varied with 68 patients (34.9%) ordered for further investigations, and 48 patients (24.6%) prescribed oral contraceptive pills (OCPs). Moreover, management of 74 patients (37.9%) included medications other than OCPs, and 17 patients (8.7%) underwent surgical intervention. Referral to other departments was documented in 42 patients (21.5%). Lastly, 19 patients (9.7%) received no documented management.

**Table 2:** Patient complaint, duration, diagnosis, and management * Patients may present with multiple complaints, diagnoses, and management approaches

Variables*	n (%)
Complaint	
Dysmenorrhea	19 (9.7%)
Menorrhagia	27 (13.8%)
Amenorrhea	30 (15.4%)
Oligomenorrhea	22 (11.3%)
Irregular cycle	51 (26.2%)
Hirsutism	13 (6.7%)
Skin rash	10 (5.1%)
Breast related (mass, pain)	5 (2.6%)
Abdominal/pelvic pain	22 (11.3%)
Vaginal symptoms (itching/discharge)	7 (3.6%)
Dysuria	1 (0.5%)
Others	42 (21.5%)
Duration period	
Since menarche	12 (6.2%)
<6 months	32 (16.4%)
6 months - 1 year	26 (13.3%)
>1 year	14 (7.2%)
Not stated	111 (56.9%)
Diagnosis categories	
Primary dysmenorrhea	5 (2.6%)
Ovarian failure	4 (2.1%)
Polycystic ovary syndrome (PCOS)	76 (39%)
Ovarian mass/cyst	22 (11.3%)
Uterine mass	4 (2.1%)
Abnormal uterus (bicornuate, absent)	4 (2.1%)
Anemia	3 (1.5%)
Endometriosis	3 (1.5%)
Abscess	1 (0.5%)
Other	51 (26.2%)
Undiagnosed/missing diagnosis	29 (14.9%)
Management plans	
Further investigations	68 (34.9%)
Oral contraceptive pill (OCP)	48 (24.6%)
Other medications	74 (37.9%)
Surgical intervention	17 (8.7%)
Referral	42 (21.5%)
Other	27 (13.8%)
No management	19 (9.7%)

A statistically significant association was found between body mass index (BMI) categories and a diagnosis of polycystic ovary syndrome (PCOS) (p<0.001) (Tables 3, 4).

**Table 3:** Correlation between BMI and PCOS diagnosis PCOS: polycystic ovary syndrome

Body Mass Index (BMI) category	PCOS: Yes (n)	PCOS: No (n)	Total (n)
Underweight	2	23	25
Normal	23	54	77
Overweight	28	24	52
Obese	23	18	41
Total	76	119	195

**Table 4:** Pearson Chi-square test for association between BMI category and PCOS status PCOS: polycystic ovary syndrome; df: degrees of freedom

Statistic	Value	df	Asymptotic significance (2-sided)
Pearson Chi-square (χ2)	22.658	3	0.000

## Discussion

This cross-sectional study provides insights into the morbidity patterns among young, unmarried Saudi women attending a gynecology clinic at King Abdulaziz Medical City in Riyadh. By focusing on a specific demographic (14-25 years, unmarried), the study addresses a gap in the literature concerning gynecological health in this often-understudied population segment within Saudi Arabia. The findings illuminate prevalent conditions, common complaints, and current management strategies, offering a foundation for targeted public health interventions and clinical practice improvements.

The study population's mean age of 19.59±3.72 years and mean BMI of 25.63±6.74 kg/m2 reflect a demographic that is transitioning from adolescence to young adulthood, a critical period for the onset of many gynecological conditions. The BMI distribution, showing a significant proportion of overweight and obese individuals, is consistent with the rising global trend of obesity, particularly in the Middle East, which has significant implications for reproductive health. This aligns with national data indicating increasing rates of overweight and obesity among adolescents and young adults in Saudi Arabia [16].

A notable finding was that over half of the participants (56.41%) presented with at least one medical condition, and more than a third (34.9%) were on chronic medication. While the specific conditions are not detailed in the provided abstract, this highlights the importance of a holistic approach in gynecological consultations, considering systemic health alongside reproductive concerns. The significant majority of patients, 146 (74.9%), reported no prior gynecological illness, which suggests that for many, the clinic visit was their initial encounter with a gynecological complaint, underscoring the role of such clinics as primary points of care for emerging issues in this age group.

Polycystic ovary syndrome (PCOS) emerged as the most prevalent diagnosis, both among those with a positive gynecological history (63.27%) and as the predominant diagnosis within the entire sample (39%). The high prevalence of PCOS in this study aligns with observations from other studies in Saudi Arabia and the broader Middle East. It is consistent with global trends where PCOS affects 5-10% of reproductive-aged women, but some regional studies have reported even higher rates, possibly due to genetic predispositions, lifestyle factors, and diagnostic criteria variations [17-19]. For instance, a systematic review of PCOS prevalence in the Middle East and North Africa region noted a wide range, with some studies in Saudi Arabia indicating high prevalence, particularly among those seeking medical attention for related symptoms [20,21]. For example, research has consistently shown that PCOS is a leading cause of infertility and metabolic dysfunction in the region, often exacerbated by lifestyle factors [22]. The strong correlation between BMI and PCOS is also well-documented globally, with obesity being a major contributor to the severity and manifestation of PCOS symptoms.

The strong association found between BMI categories and PCOS diagnosis (p<0.001) further reinforces the well-established link between obesity and PCOS pathophysiology, where insulin resistance plays a central role [23]. For example, a study done by Barber et al. revealed a correlation between PCOS and abnormal BMI that echoes this study’s findings [24]. Similarly, Neubronner et al. found that women with PCOS have a higher mean BMI and high BMI’s association with exacerbated features of PCOS like hirsutism [25]. This emphasizes the need for early screening and lifestyle interventions targeting weight management in this vulnerable population.

Menstrual irregularities were the most frequently reported complaints, with irregular menstrual cycles (26.2%), amenorrhea (15.4%), and menorrhagia (13.8%) being the top three. These complaints are often hallmarks of underlying endocrine imbalances, including PCOS, and can significantly impact quality of life. The high incidence of these symptoms necessitates thorough investigation and appropriate management. The substantial proportion of undocumented complaint duration, in 111 cases (56.9%) represents a significant data gap, potentially hindering comprehensive clinical assessment and epidemiological understanding. This highlights a need for improved documentation practices in electronic medical records.

The reported patterns of menstrual irregularities (irregular cycles, amenorrhea, menorrhagia) are common gynecological complaints worldwide, particularly in adolescent and young adult populations. However, the specific distribution and their association with underlying diagnoses like PCOS provide context for the Saudi population. Studies from other countries also report high rates of menstrual irregularities, often linked to lifestyle, stress, and underlying endocrine disorders [26].

Management strategies varied, with a significant emphasis on further investigations in 68 cases (34.9%), reflecting the diagnostic complexity often associated with gynecological complaints in young women. The prescription of oral contraceptive pills (OCPs) in 48 cases (24.6%) and other medications in 74 cases (37.9%) indicates a reliance on pharmacological approaches, which are standard for managing menstrual irregularities and PCOS symptoms. The use of OCPs was the third most common approach to managing patients, which is logical as PCOS was the most common diagnosis. OCP is established as the first line treatment of PCOS in women who are not seeking pregnancy at the time [27], as OCP manages hirsutism and acne faced by women with PCOS [28]. Surgical intervention in 17 cases (8.7%) and referrals to other departments for 42 cases (21.5%) point to the multidisciplinary nature of care required for some conditions. A notable finding was that 19 patients (9.7%) had no documented management plan. This absence of documentation is concerning and necessitates further investigation to ascertain the underlying reasons, which may include patient non-compliance, incomplete record-keeping, or deferred clinical decision-making

Lastly, studies have previously shown there to be a stigma against seeking gynecological care, under the assumption that it is always related to pregnancy or sexually transmitted diseases (STDs) [29,30]. By showcasing the most common gynecological morbidities faced by young, unmarried women to be neither pregnancy nor STDs, this study hopes to aid in removing the stigma surrounding gynecological care for young unmarried women and encourage unmarried women to attend gynecological clinics in Riyadh, Saudi Arabia.

Strengths and limitations

A key strength of this study is its focus on a specific, often overlooked, demographic: unmarried Saudi women aged 14-25 years. This provides unique insights into their gynecological health needs. The use of electronic medical records (BestCare) allowed for the systematic collection of a relatively large dataset over a five-year period, enhancing the study's robustness.

However, several limitations must be acknowledged. As a cross-sectional, single-center study, its findings may not be generalizable to the entire Saudi female population or to other healthcare settings. The retrospective nature of data collection from electronic medical records is subject to potential biases, such as incomplete or inaccurate documentation, as evidenced by the high percentage of undocumented complaint durations. Furthermore, the study design does not allow for the establishment of causality between factors like BMI and PCOS, only association.

## Conclusions

This study described the most common morbidities among young unmarried women visiting gynecological clinics, and the significant prevalence of PCOS and menstrual irregularities. The findings emphasize the need for targeted clinical approaches, robust health education, and improved data collection to address the unique gynecological health challenges faced by this demographic in Saudi Arabia. Future research should consider prospective, multi-center studies to validate these findings and provide a more generalized understanding of gynecological morbidity in Saudi Arabia.
